# Switch from premixed insulin analogue to degludec-liraglutide combination: a CGM study

**DOI:** 10.3389/fendo.2025.1715800

**Published:** 2025-11-05

**Authors:** Nika Aleksandra Kravos Tramšek, Andrijana Koceva, Mitja Krajnc

**Affiliations:** ^1^ Department of Endocrinology and Diabetology, University Medical Center Maribor, Maribor, Slovenia; ^2^ Faculty of Medicine, University of Maribor, Maribor, Slovenia

**Keywords:** diabetes mellitus, premixed insulin, GLP-1 analogue, IDegLira, continuous glucose monitoring (CGM), CGM targets

## Abstract

**Introduction:**

Basal insulin with glucagon-like peptide-1 receptor agonist could be preferred over premixed insulin for intensification in type 2 diabetes due to better glycemic control, lower hypoglycemia risk, and favorable effects on body weight. Comparative data on premixed insulin and the combination of degludec and liraglutide (iDegLira) are limited.

**Methods:**

We conducted a 24-week single-arm prospective study to evaluate the impact of iDegLira compared to premixed insulin on the regulation of diabetes and glucovariability using continuous glucose monitoring (CGM), HbA1c, and anthropometric measurements. A total of 37 participants with type 2 diabetes (20 male and 17 female, aged 70.2 ± 10.0 years with BMI 31.0 (28.0-34.0) kg/m^2^ and duration of diabetes for 15.2 ± 7.7 years) were switched from premixed insulin treatment to iDegLira. The primary outcome was the change in HbA1c. Secondary outcomes included change in time in range (TIR) from baseline to 6 months, change in time below range (TBR), change in nocturnal hypoglycemia, glucovariability, insulin dose and body weight.

**Results:**

We observed improved glycemic control on iDegLira with improvement of average fasting glucose (6.92 ± 1.64 *vs*. 8.25 ± 2.2 mmol/l; p<0.031), HbA1c (7.10 ± 0.7% *vs*. 7.39 ± 0.7% p=0.045) and TIR (71.2 ± 17.2% *vs*. 64.3 ± 18.0; p=0.027). These results were accompanied by a nearly halved total daily insulin dose (-21 units/day, p<0.001) and a modest reduction of body weight.

**Discussion:**

iDegLira improved glycemic control, resulting in a lower HbA1c and higher TIR, alongside beneficial effects on body weight and total daily insulin doses. While numerical reductions in hypoglycemia did not reach statistical significance, treatment was not associated with an increased risk of hypoglycemia. iDegLira can be an efficient and safe treatment option, providing simplified treatment with improved glycemic control.

## 1 Introduction

Type 2 diabetes mellitus is a chronic disease that is estimated to affect 510.8 million people by 2030. Based on projections, global insulin consumption is expected to rise from 516.1 million 1,000 IU vials annually in 2018, to 633.7 million vials annually by 2030 (1). Guidelines recommend adding glucose-lowering treatments step by step according to HbA1c to control glycemia, which significantly impacts the frequency of complications associated with the disease (2). With the development of sodium-glucose co-transporter-2 inhibitors and glucagon-like peptide-1 receptor agonist (GLP-1 RA) we have more treatment options, including drugs that not only improve glycemic control but also reduce cardiovascular and renal complications (3). According to the guidelines, GLP-1RA is the first treatment option when introducing injectable therapy, followed by insulin treatment combinations, such as basal insulin or premixed insulin and fixed-ratio combination (FRC) of basal insulin and GLP-1 RA, such as the combination of degludec and liraglutide (iDegLira) or glargine with lixisenatide (iGlarLixi) (2). The main problem of introducing injectable therapy is clinician inertia, which includes resistance to both the introduction of injectable therapy and insulin titration. Consequently, insulin therapy can be delayed for more than 6 years, leading to poor glycemic control and increased complications (4). Premixed insulin can improve glycemic control but can increase the risk of hypoglycemia and body weight gain, which can lead to poor adherence (4). Premixed insulin is usually considered for older patients who are unwilling or unable to adhere to a basal-bolus scheme (5). With the development of more stable insulin analogues and FRC, we have more options for personalized treatment of diabetes mellitus.

Recent studies suggest that FRC of basal insulin with GLP-1 RA is a valid treatment option, especially for patients with frequent hypoglycemic events or difficulties maintaining weight (6). Retrospective studies comparing iGlarLixi and the basal-bolus scheme resulted in higher persistence, adherence, and lower hypoglycemia on FRC, although the reduction in HbA1c was lower (7). The iDegLira showed similar HbA1c reduction to basal-bolus therapy but with significantly less hypoglycemia and weight gain, and comparable or superior results compared to monotherapy with insulin degludec or liraglutide (8). None of these studies evaluated glycemia with continuous glucose monitoring (CGM). Liraglutide significantly reduces body weight by 4.7-6.0%, depending on the dose, with weight loss greater than 5% occurring in 40.4-54.3% of patients (9). iDegLira was associated with greater weight reduction and lower insulin requirements, whereas iGlarLixi provided superior postprandial glucose control in real-world comparative studies (10).

To our knowledge, only one study has directly compared FRC iDegLira with premixed insulin. The results showed that higher insulin doses improved both fasting and postprandial glycemia, however, these benefits were accompanied by an increase in body weight due to greater insulin requirements. In contrast, treatment with iDegLira improved fasting and postprandial glycemia as well as HbA1c, while also being associated with a reduction in body weight (11).

Extensive evidence supports the clinical advantages of CGM across different insulin therapies, including in individuals with type 2 diabetes (12). However, data on CGM use among patients receiving premixed insulin remain limited (2). Within CGM-derived metrics, optimal and safe glycemic premixed is primarily characterized by time in range (TIR) above 70%, and time below range (TBR) below 5%. The recommended target for glycemic variability, expressed as the coefficient of variation (CV), is ≤ 36% (12).

Our study aimed to investigate the impact of premixed insulin versus FRC iDegLira on anthropometric parameters, HbA1c, TIR and the daily glucose fluctuation measured with CGM. This is the first study to use CGM in that setting.

## 2 Materials and methods

### 2.1 Participants and study design

We conducted a prospective study including 37 participants treated in the diabetology outpatient clinic at the University Medical Center Maribor. Inclusion criteria included known diagnosis of type 2 diabetes mellitus for at least six months, treatment with two daily doses of premixed insulin either premixed aspart 30 (NovoMix(30) by NovoNordisk) or premixed lispro 25 (HumalogMix(25) by Eli Lilly), age over 18 years, HbA1c below 10% and a total daily insulin dose of less than 70 units. Exclusion criteria were the need for higher doses of insulin, poorly controlled diabetes with HbA1c above 10%, end-stage renal disease, pregnancy or breastfeeding and current use of a concomitant medication that could negatively affect glycemia or sensor accuracy (glucocorticoids, hydroxyurea, high doses of paracetamol). All patients regularly received diabetes self-management education and support. All participants signed the informed consent. Our institution’s medical ethics committee approved the study with No. UKC-MB-KME/64/21.

The patients received a blinded CGM system for 10 days at baseline while still on premixed insulin therapy. Then they were switched to FRC of iDegLira basal insulin degludec and GLP-1 RA liraglutide (iDegLira; 50 units of degludec/1.8 mg liraglutide) with a starting dose of 16 dose steps and titrated to fasting glucose targets of 5–7 mmol/L. After 6 months of iDegLira treatment, we repeated anthropometric measurements, laboratory tests, and application of CGM for 10 days.

### 2.2 Research methods

This study aimed to evaluate the impact of switching from premixed insulin to the FRC iDegLira on glycemic control and glucovariability, assessed by CGM, HbA1c, and anthropometric measurements. As this was a non-randomized, self-controlled study, each participant served as their own control, allowing within-subject comparisons. Data on age, sex, diabetes duration, daily dose of insulin, concomitant medications, and known diagnosis of micro- and/or macrovascular complications were collected at baseline and at the end of the study. Measured anthropometric parameters included body weight in kilograms, body height in centimeters, waist circumference in centimeters and calculated body mass index (BMI is body weight divided by the square of body height). Fasting glucose and HbA1c were assessed at baseline and after 6 months. A real-time Dexcom G6 personal CGM system (Dexcom, Inc., San Diego, California) was used as it does not need calibration and can be worn for up to 10 days (13, 14). We used devices in a blinded fashion. The Dexcom G6 sensor was applied by a trained nurse educator using the standard auto-insertion device, and participants received instructions regarding appropriate sensor maintenance. Each sensor remained in place for up to 10 days unless it was removed earlier due to technical issues or participant preference. Data were processed with the dedicated Dexcom Clarity software. CGM metrics included: percentage of time in range (TIR), time below range (TBR; 3.0–3.8 mmol/L and <3.0 mmol/L), time above range (TAR; 10–13.9 mmol/L and >13.9 mmol/L), coefficient of variation (CV%) as an index of glycemic variability, and the glucose management indicator (GMI). In addition, peak postprandial glucose levels after breakfast, lunch, and dinner (within 3 hours of meal intake) were visually identified from Dexcom Clarity graphs, using participants’ self-reported mealtimes. The software also enabled quantification of the proportion of TBR occurring overnight (00:00–06:00), representing nocturnal hypoglycemia. The patients were instructed to maintain their daily routines and adhere to their prescribed medications.

### 2.3 Statistical analysis

In order to detect a mean difference of ≥ 0.5% in HbA1c with 90% power (α =0.05) and a standard deviation (SD) of 0.8%, 27 individuals were required for a paired t-test. To detect a 10-percentage-point difference in time in range (TIR) with 90% power and an SD of 18-percentage-points, 34 participants were required. To account for potential attrition and incomplete CGM data, 37 individuals were recruited.

The Shapiro-Wilk test was used to assess the normality of variable distributions and the distribution of differences. Normally distributed variables were described using the mean and standard deviation, while non-normally distributed variables were reported using the median and interquartile range.

To compare data between treatments, we used the paired Student’s t-test when the assumption of normality was met. Otherwise, we applied the Wilcoxon signed-rank test. We also calculated 95% confidence intervals.

Statistical analysis was conducted using the latest open-source software, Jamovi (2.6.2), available online at https://www.jamovi.org. A p-value < 0.05 was considered statistically significant.

## 3 Results

The study included 37 patients, 20 male and 17 female, aged 70.2 ± 10.0 years with BMI 31.0 (28.0-34.0) kg/m2. The average duration of diabetes was 15.2 ± 7.7 years. In the last 6 months, 30 (81%) have been treated with premixed aspart and 7 (19%) with premixed lispro. Concomitant medications included metformin (70.2%) and sodium-glucose co-transporter 2 inhibitors (37.8%). Two-thirds of participants (62.1%), had at least one microvascular complication. Baseline characteristics are summarized in **Table 1**.

**Table 1:** Baseline characteristics.

Characteristic	Value
Age	70.2 ± 10.0
Sex (female/male)	17/20
Diabetes duration (years)	15.2 ± 7.7
HbA1c (%)	7.39 ± 0.72
Body weight (kg)	86.5 ± 14.3
Body mass index (BMI) (kg/m^2^)	31.0 (28.0-34.0)
Insulin dose (units/day)	44.5 ± 12.5

Average fasting glucose levels on premixed insulin were 8.25 ± 2.2 mmol/l and after 6 months of FRC iDegLira were 6.92 ± 1.64 mmol/l (p<0.031). Both HbA1c and TIR were significantly improved after the treatment switch with p=0.045 and p=0.027, respectively. The occurrence of hypoglycemic events was not significantly different between premixed insulin and FRC. CV does not differ between therapies, with values of 28.6% and 27.5%, respectively, but both values are already below the recommended threshold of less than 36%.

We also observed an impact on insulin requirement and on body weight. The average insulin dose in premixed insulin was 44.5 ± 12.5 units/day, which was reduced significantly on FRC iDegLira with 23.1 ± 8.9 units/day (p<0.001). The introduction of GLP-1 RA in FRC significantly impacted body weight, with an average reduction of 2 kg in 6 months (p>0.001). There was no difference in metformin use, SGLT-2 inhibitors, or the presence of microvascular complications at baseline and after 6 months. The main findings are shown in **Table 2**.

**Table 2:** Results of parameters on premixed insulin versus iDegLira.

Parameter	Baseline on premixed insulin	After 6 months on iDeg/Lira	P-value
TIR (%)	64.3 ± 18.0	71.2 ± 17.2	**0.027**
TBR < 70 mg/dl (%)	0.0 (0.0-1.0)	0.0 (0.0-1.0)	0.203
TBR < 54 mg/dl (%)	0.0 (0.0-0.0)	0.0 (0.0-0.0)	0.095
Nocturnal hypoglycemia < 70	1.0 (0.0-2.0)	0.0 (0.0-0.0)	0.099
Nocturnal hypoglycemia < 54	0.0 (0.0-0.0)	0.0 (0.0-0.0)	0.136
CV (%)	28.6 ± 7.0	27.5 ± 5.4	0.229
GMI	7.44 ± 0.8	7.09 ± 0.7	**0.021**
HbA1c (%)	7.39 ± 0.7	7.10 ± 0.7	**0.045**
Fasting glucose level	8.25 ± 2.2	6.92 ± 1.64	**0.031**
Daily insulin dose (units/day)	44.5 ± 12.5	23.1 ± 8.9	**<0.001**
Body weight (kg)	86.5 ± 14.3	84.5 ± 13.6	**<0.001**
Body mass index (BMI) (kg/m^2^)	31.0 (28.0-34-0)	30.0 (27.0-34.0)	**0.002**

Bold values indicate results that are statistically significant.

## 4 Discussion

In our study, we demonstrated that switching from premixed biphase insulin treatment to a combination of iDegLira for six months may improve glycemic control, reduce insulin requirements, and promote weight loss in patients with type 2 diabetes, while maintaining low glycemic variability.

Our study indicates that treatment with iDegLira FRC significantly improves glycemic control as defined by TIR and HbA1c. TIR provides a comprehensive assessment of daily glycemic control and fluctuation, making it more accurate in predicting glycemic control improvement than HbA1c. HbA1c reflects mean glucose levels over a three-month period and can be influenced by other factors, whereas TIR captures short-term glucose variability. Therefore, incorporating TIR alongside or in place of HbA1c enhances individualized diabetes management by providing a dynamic and patient-centered evaluation of glycemic control. The 7% increase in TIR exceeded the clinically relevant threshold of 5% as defined by the ATTD consensus (12), corresponding to approximately one additional hour per day spent in the target range associated with a risk reduction of microvascular complications. Fasting glucose levels and GMI also showed significant decreases.

It should be noted that participants in our study were already relatively well controlled at baseline (mean HbA1c ≈ 7.4%). This may have limited the magnitude of improvement observed and could underestimate the potential benefits of iDegLira in individuals with poorer baseline glycemic control.

Although TBR and nocturnal hypoglycemia decreased numerically, the differences did not reach statistical significance. This is likely due to the low baseline frequency of hypoglycemic events, the relatively short CGM observation period, and the limited sample size, reducing the power to detect rare outcomes.

The reduction in injection frequency from two to one per day may also have contributed to the observed improvement, a finding consistent with results from a prospective study in older adults with type 2 diabetes. Treatment simplification using FRC has been shown to enhance Diabetes Treatment Satisfaction Questionnaire scores as well as measures of daily functioning and mental well-being. These benefits are likely attributable to improved treatment adherence and efficiency, without accompanying increases in hypoglycemia or body weight (15).

The mean HbA1c level decreased by 0.3%. This modest reduction is meaningful and clinically relevant as it was achieved with a substantially reduced insulin dose and without an increase in hypoglycemia. The daily insulin dose was almost halved (-21 units/day, p<0.001), which may also be associated with a lower risk of hypoglycemia and reduced insulin-related weight gain.

In this cohort of individuals with overweight or class I obesity, we observed a 2 kg average reduction in body weight, corresponding to a 2.3% decrease from baseline body weight, as well as a decrease in BMI of 1 kg/m². Although this body weight reduction is modest, it is clinically favorable in insulin therapy, which is usually associated with weight gain (16).

We did not observe a significant change in glycemic variability (from 28.6% at baseline to 27.5% after six months, p=0.237). Both values are already below the recommended threshold of less than 36%, indicating that patients had stable glycemia prior to treatment switch and the intervention did not increase variability. Improved glycemic stability is clinically relevant, since variability has been identified as an independent predictor of diabetes complications. Glycemic control significantly reduces the risk of micro- and macrovascular diabetes complications, especially mortality, renal disease and retinopathy (2, 17). These findings align with prior evidence showing that GLP-1 receptor agonists contribute not only to weight reduction and improved glucose control but also to cardiovascular protection, reducing MACE by 14% and hospital admission for heart failure by 11%, as well as renal protection, reducing the composite kidney outcome by 21% (18–21).

We have observed that there are fewer episodes of hypoglycemia with FRC therapy. As shown in previous studies among insulin-based treatment strategies, FRC is associated with a lower incidence of hypoglycemia and less weight gain compared to multiple daily injections, while achieving comparable glycemic control. Once-daily FRC offers a practical approach, with straightforward dose adjustment, favorable effects on quality of life, and effective fasting glucose management (15). Inertia toward increasing the insulin dose is a known cause of worsening glycemic control (4). Reducing injection frequency to once daily and allowing flexible dosing with any main meal of the day can lessen patient resistance to initiating or intensifying insulin therapy (22). Reducing injection frequency enables patients to have a more flexible day plan, accommodating irregular schedules. It also reduces the frequency of missed doses. Despite these benefits, inertia remains a challenge that can delay achievement of glycemic targets.

The main limitations of our study are the non-randomized study design, the small sample size (n = 37), and the relatively short CGM follow-up, which limit statistical power and generalizability. Despite these limitations, the observed improvements in TIR, HbA1c, insulin dose and body weight are consistent with findings from previous randomized controlled trials (23–25).

## 5 Conclusion

In summary, our findings suggest that switching from premixed insulin to iDegLira may simplify treatment and improve glycemic outcomes, as evident by increases in TIR and reductions in HbA1c, fasting glucose, insulin dose and body weight, without increasing hypoglycemic events. Given the relatively well-controlled baseline HbA1c and TIR in our cohort, these findings may underestimate the potential benefits in less well-controlled populations. Larger, randomized studies should be prioritized to further confirm these findings and define which patient subgroups may derive the most significant benefits.

## Data Availability

The datasets presented in this article are not readily available because Data will be shared upon reasonable request. Requests to access the datasets should be directed to nikakravos@gmail.com.
